# Functionalized Fe_3_O_4_ Nanoparticles as Glassy Carbon Electrode Modifiers for Heavy Metal Ions Detection—A Mini Review

**DOI:** 10.3390/ma14247725

**Published:** 2021-12-14

**Authors:** Amanda Kulpa-Koterwa, Tadeusz Ossowski, Paweł Niedziałkowski

**Affiliations:** Department of Analytical Chemistry, Faculty of Chemistry, University of Gdansk, Wita Stwosza 63, 80-308 Gdansk, Poland; tadeusz.ossowski@ug.edu.pl

**Keywords:** magnetite nanoparticles, Fe_3_O_4_, electrode modification, electrochemical sensor, heavy metal ions detection

## Abstract

Over the past few decades, nanoparticles of iron oxide Fe_3_O_4_ (magnetite) gained significant attention in both basic studies and many practical applications. Their unique properties such as superparamagnetism, low toxicity, synthesis simplicity, high surface area to volume ratio, simple separation methodology by an external magnetic field, and renewability are the reasons for their successful utilisation in environmental remediation, biomedical, and agricultural applications. Moreover, the magnetite surface modification enables the successful binding of various analytes. In this work, we discuss the usage of core–shell nanoparticles and nanocomposites based on Fe_3_O_4_ for the modification of the GC electrode surface. Furthermore, this review focuses on the heavy metal ions electrochemical detection using Fe_3_O_4_-based nanoparticles-modified electrodes. Moreover, the most frequently used electrochemical methods, such as differential pulse anodic stripping voltammetry and measurement conditions, including deposition potential, deposition time, and electrolyte selection, are discussed.

## 1. Introduction

Nanotechnology has become a popular and rapidly developing field of science and industry since Nobel Prize winner R.P. Feynman’s breakthrough in 1959 [1]. A series of nanomaterials has been attracting researchers’ attention due to the significant features of these materials, such as excellent electrical, optical, magnetic, and catalytic properties [2]. The properties and potential applications of nanoparticles depend on their phases, sizes, and morphologies [3].

Recently, nanomaterials with magnetic properties, especially those comprising iron oxide Fe_3_O_4_, have gained considerable popularity. Magnetite (Fe_3_O_4_) nanoparticles have been widely used in many fields because of their unique electric and magnetic properties. Fe_3_O_4_ nanomaterials are found in many important applications in industrial areas, such as lithium-ion batteries [4,5], catalytic sorption [6], microwave absorption [7,8], and photocatalytic degradation [9,10,11]. Furthermore, magnetite-based nanocomposites are extensively used in biomedicine, in particular the photothermal killing of breast cancer cells [12], cell targeting and sorting, drug delivery vehicles [13,14], magnetic resonance [15,16], and fluorescence imaging [17]. Due to the increasing environmental pollution from heavy metals, nanomaterials based on magnetite are widely applied in environmental protection as metal ion adsorbents for metal ions remediation [18,19].

Magnetic Fe_3_O_4_ nanoparticles have been used as a basis for the development of many synthesis methods. There are plenty of Fe_3_O_4_ synthesis methods, including coprecipitation, sonochemical reaction, hydrothermal reaction, microemulsion and sol-gel synthesis, and cathodic electrochemical deposition [14,20,21,22,23]. Interestingly, an important characteristic of nanomagnetite is its surface modification ability, which increases its applicability [24]. The majority of synthesis methods are simple and quick in preparation. Furthermore, there are many synthetic methods that can be used to obtain different nanoparticle sizes [24,25,26,27,28] and shapes [28,29,30]. Nanomagnetite can be obtained in various sizes and shapes, including the most popular spherical nanoparticles and in cuboids, octahedrons, plates, tetrahedrons, concaves, octapods, multibranches, and nanorods [31,32,33,34]. Fe_3_O_4_ nanoparticles are the basic material for subsequent surface modifications creating core–shell structures, which affect the further extension of their applications in many fields. 

**Figure 1:**
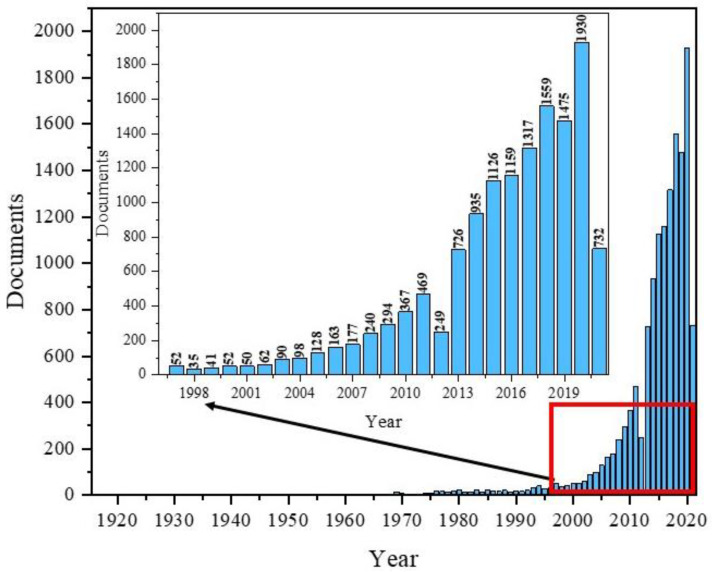
Scheme of the number of publications with “Fe_3_O_4_” in title based on the year of publication using the Scopus base.

To the authors’ knowledge, the very first paper covering Fe_3_O_4_ was published in 1916 by the Americans, Sosman and Hostetter, and focused on iron oxides in general [35]. Over the next few decades, several articles appeared each year. Since the 1990s, we have observed a growing interest in nanomagnetites. The highest number of publications in the field with “Fe_3_O_4_” in the title appeared in 2020, totalling 1930 papers (Figure 1).

The data presented in Figure 1 shows the popularity of Fe_3_O_4_ and its dynamics as a topic of publication, especially during the most recent years. Additionally, composites based on Fe_3_O_4_ are used in many fields of science and industry, including magnetic separation, magnetic catalysis, environmental treatment, food analysis, target drug delivery systems, biosensors, magnetic resonance imagining, hyperthermia, and tissue engineering [36].

There are plenty of magnetite nanoparticles and hybrid structures used in the electrochemical detection of heavy metal ions providing an excellent basis for further functionalisation. The surface of Fe_3_O_4_ nanoparticles can be combined with nanoparticles of other metals (Au [37]); oxides (SiO_2_ [38,39] and TiO_2_ [40]) and additional conductive materials (GO [41]); complicated, organic functional groups (dendrimers [42] and polymers [43]); and biological particles fragments (DNA [44]). Fe_3_O_4_-based nanomaterials possess a high adsorption capacity, which makes them suitable for the electrochemical detection of metals [45].

The Fe_3_O_4_ nanoparticles are easy to oxidise and aggregate, which results in their low magnetic properties [46]; therefore, there is a need to coat bare nanomagnetite with polymer or inorganic shells. Additionally, the modification can increase the biocompatibility of these material [47].

In this work, we describe the recently published applications of a variety of functionalised Fe_3_O_4_ nanoparticles to electrode surface modifications to create a sensor for heavy metal ions. We discuss the preparation procedure of GCE for a sensor and the methods of electrode modification using Fe_3_O_4_ nanoparticles. We also present the most frequently used electrochemical techniques with their characteristic measurement parameters. Finally, we present a performance comparison of the recently developed heavy metal ion sensors.

## 2. Nano-Fe_3_O_4_ as Electrode Modifiers

Based on several decades of intensive research, Fe_3_O_4_ has become one of the best characterised metal oxides. Its cubic crystallographic system contains both Fe^3+^ and Fe^2+^ ions. Fe_3_O_4_ is a black solid with a density of 5.18 g·cm^−3^, Mohs hardness of 5, melting point range of 1583–1597 °C, and boiling point of 2623 °C. Its characteristic magnetic feature is the ferrimagnetism at room temperature and Neel (Curie) temperature of 850 °C [48].

In the past few years, Fe_3_O_4_ nanoparticles have become a focus of interest for numerous scientific groups. In the nano range (in diameter from 1 to 100 nm) smaller than 6 nm, magnetite particles indicate superparamagnetic properties, although their magnetic features strongly depend on the synthesis method [49]. Based on the gathered evidence, the nanomagnetites in most applications show the best characteristics in the range of 10–20 nm. Decreasing the nanoparticles’ size leads to an increase in the specific surface. Furthermore, the nanoparticles’ size strongly influences their magnetic moment and reaction to the magnetic field and depends on their size and shape [50]. Electrochemistry, and electrode modifications for the generation of a highly sensitive sensor, is one of the most rapidly developing fields of science. Electrochemical sensing is focused on the development of new electrode materials with better properties compared to commercial electrodes. The perfect sensor should exhibit a signal output proportional to the number of target species, high selectivity, sensitivity, repeatability, and rapid response [50].

**Figure 2:**
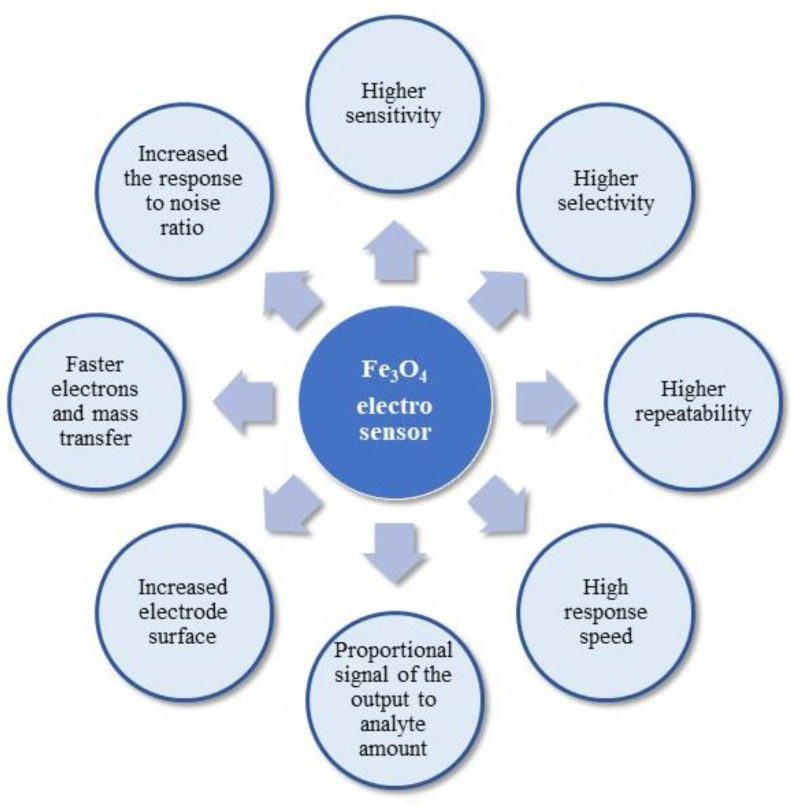
Scheme of the important electro-sensor features.

Nanomagnetite has been widely employed as a promising modifier due to its unique properties, low-cost, easy preparation, non-toxicity, excellent absorption capacity, catalytic properties, and inherent electrical conductivity [51]. The electrochemical performance of an electrode is closely related to the absorption capacity and the conductivity of the modified material. The imposition of Fe_3_O_4_ nanocomposites on the electrode surface causes the enhancement of the electrode area, enhancement of the rate of mass and electron transfer, improved selectivity and sensitivity, and, most importantly, increased response to the noise ratio [50]. Furthermore, Fe_3_O_4_-based electrochemical detection systems are characterised by small dimensions, costlessness, sensitivity, flexibility, and quickness in use [52]. The advantages of Fe_3_O_4_ usage as an electro-sensor are described in Figure 2.

## 3. Recent Electrode Modifications with Magnetic Nanoparticles to Heavy Metal Ions Detection

The glassy carbon (GC) electrode is the most commonly used electrode for electroanalytical purposes due to its unique electrical conductivity, chemical stability, biocompatibility, and wide potential range and extremely low gas permeability [53]. Therefore, the GC electrode is an excellent material for modification to obtain a stable surface used as a biosensor. First of all, sensor development requires proper preparation of the electrode for further modification. Before each modification, the GC electrode usually needs to be polished to a shiny, mirror-like surface with wet alumina slurry—Al_2_O_3_ powder of different sizes, 1.0 μm, 0.3 μm, and 0.05 μm, using a polishing cloth and rinsing with water. Then, successive washing or sonications in absolute ethanol and ultrapure water are usually conducted, sometimes in a 1:1 (*v*/*v*) HNO_3_ solution, lasting at least a few minutes each [54]. Subsequently, the electrode surface is dried with nitrogen or at room temperature, and the electrode is ready for further use. As an exception, Miao et al. started the modification with GCE soaking in piranha solution (98% H_2_SO_4_:30% H_2_O_2_ = 3:1) for about 5 min to remove any adsorbed materials [44].

**Figure 3:**
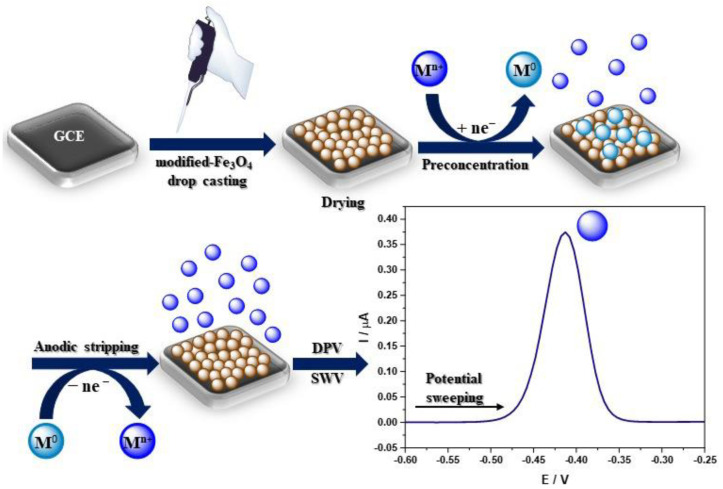
Schematic visualisation of the sensor development and heavy metal ions electrochemical detection.

A homogeneous suspension of nanoparticles is necessary to modify the electrode, most often by sonication in deionized water, absolute ethanol, and sometimes an ethanol solution containing 0.25 wt% Nafion^®^ [55] or IPA [56], or DMF [57] in a concentration of 1 mg/mL. Ultrasonic bath sonication lasts from 5 min to 2 h, but most often 30 min or until a uniform suspension is obtained. The most common method of electrode modification is drop-casting while the nanoparticles’ suspension is pipped on the electrode surface (Figure 3) [58]. The amount of applied nanoparticles depends on the active surface of the working electrode. After the modification, the electrode was dried at room temperature until the solvent completely evaporated, which usually takes from several minutes to a few hours. A different approach was presented by Kong et al., where 6 mL of Fe_3_O_4_@PANI nanoparticles suspension was pipetted onto an electrode, and after drying at 4 °C in a refrigerator, the electrode was coated with 3 mL of Nafion^®^ solution (0.5 wt%) [59]. Moreover, Wang et al. created an unconventional sensor by adding the Fe_3_O_4_@PDA@MnO_2_ NPs homogenous suspension to the HCl solution (pH 3.0) with various concentrations of Pb^2+^. Then, nanoparticles with already adsorbed Pb^2+^ ions were completely transferred onto the mGCE for immediate electrochemical measurements (Figure 3) [60]. Recent findings concerning heavy metal ion detection with the GC electrode modified using Fe_3_O_4_-based nanocomposites are presented in Table 1. The authors focused only on reports published in the last 5 years related to heavy metal ions’ electrochemical analysis of GC electrodes modified with Fe_3_O_4_-based nanocomposites (Table 1).

Electrochemical techniques, especially voltammetry, include electroanalytical methods for the determination of one or more analytes by measuring the current as a function of the potential. There are a few component techniques used to obtain information on the analyte, including CV, DPV, SWV, and stripping voltammetry [70]. Voltammetric techniques are widely used in heavy metal ions detection due to their precision and sensitivity. The most frequently chosen are DPV or alternative SWV techniques (Table 1.) due to their high sensitivity and lower detection limits, which are suitable for trace level analysis. However, square wave voltammetry is preferable for obtaining the response rate. The most frequently used method for quantitative analysis is stripping voltammetry. There are two types of stripping voltammetry, ASV and CSV, depending on the chosen concentration potential [71]. The two steps of stripping analysis include analyte deposition at the electrode surface (or in its volume, e.g., HDME [72]) and analyte quantification by potential sweeping [73]. During stripping experiments, a certain voltage is applied to the GC electrode to reduce the metal ions on the electrode surface into the elemental metal, following which linear voltammetry is performed from negative to positive to oxidate the preconcentrated metal back into ions (Figure 3). The ions detection is determined according to the oxidation current produced by the process. According to the literature, the ions detection mechanism can be illustrated with the following equations (M, metal; n, number of exchanged electrons) (Figure 3) [59]:

ne^−^ + M^n+^ → M^0^ (1)

M^0^ → M^n+^ + ne^−^ (2)

**Figure 4:**
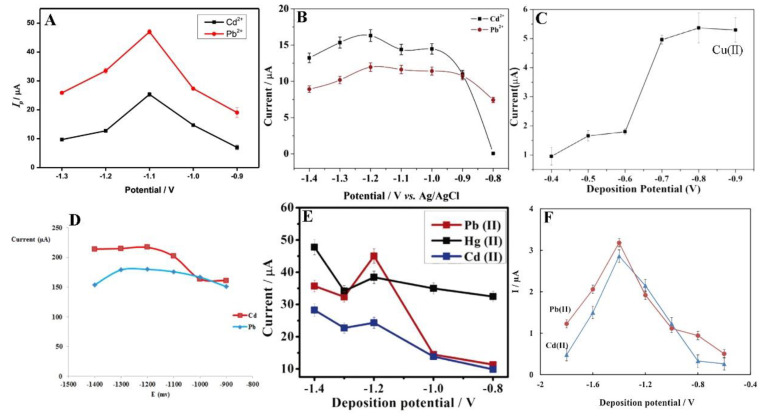
Optimisation of deposition potential used in electrochemical determination of different ions such as Cd^2+^, Pb^2+^, and Hg^2+^ presented by Pu et al. [61] (**A**), Xu et al. [63] (**B**), Wei et al. [55] (**C**), Dahaghin et al. [66] (**D**), Deshmukh et al. [68] (**E**), and Baghayeri et al. [69] (**F**). All Figures are adapted from references [55,61,63,66,68,69] with permission from Elsevier.

In the stripping analysis, the most significant parameters are potential and time of accumulation. Deposition potential should be slightly lower than the oxidation potentials of analytes. Obviously, each experiment is preceded by the optimisation of the measurement conditions. Nevertheless, in the case of metal ions, the anodic range with an optimum potential of −1.2 V, and sometimes lower to −1.4 V, is most commonly used [65,69]. Moreover, with an accumulation potential more negative than −1.2 V, a decrease in the current intensity was observed (Figure 4) [66]. The current intensity weakening can be attributed to the H_2_ evolution that deteriorates the working electrode surface activity [64,68].

**Figure 5:**
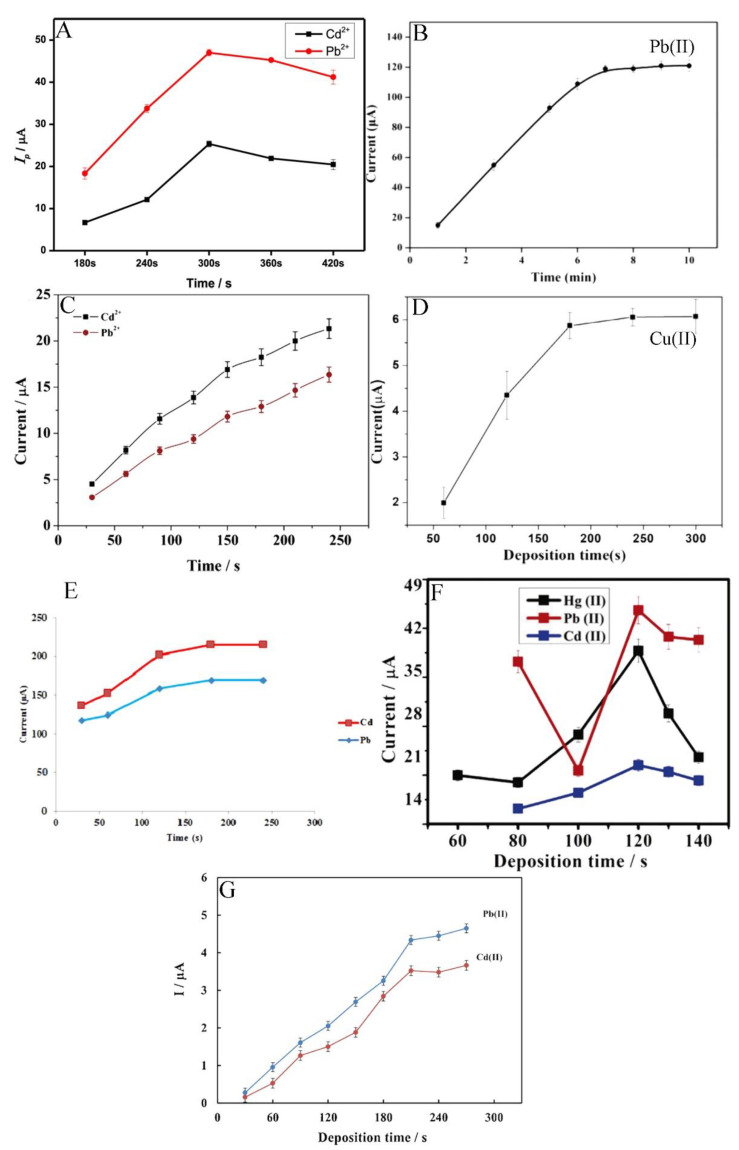
Optimisation of deposition time of different ions such as Cd^2+^, Pb^2+^, and Hg^2+^ determination by electrochemical methods presented by Pu et al. [61] (**A**), Wang et al. [60] (**B**), Xu et al. [63] (**C**), Wei et al. [55] (**D**), Dahaghin et al. [66] (**E**), Deshmukh et al. [68] (**F**), and Baghayeri et al. [69] (**G**). All Figures are adapted from references [55,60,61,63,66,68,69] with permission from Elsevier.

The metal ions’ electro-reduction time on the electrode surface starts from 120 s and reaches up to 480 s (Figure 5) [54,60]. However, the most common concentration time is within 180 s. For example, Pu et al. examined the effect of accumulation times within the range of 180 s to 420 s and observed that the peak currents of Cd^2+^ and Pb^2+^ increase linearly as the deposition time increases from 180 s to 300 s, after which the peak currents achieved plateau. Consequently, the deposition time of 300 s was used in all subsequent experiments [61]. This is caused by the saturation of selective sites on the electrode surface with the ions. Because of this phenomenon, the electrode surface does not tend to absorb more species [64]. 

Additionally, after the stripping experiment, Fan et al., Wu et al., and Pu et al. introduced heavy metal ions oxidation in the measurement method to remove the residual metals and clean the electrode surface by applying the desorption potential: 0.9 V for 150 s, 1.0 V for 210 s, and 0.2 V for 120 s [54,61,62].

**Figure 6:**
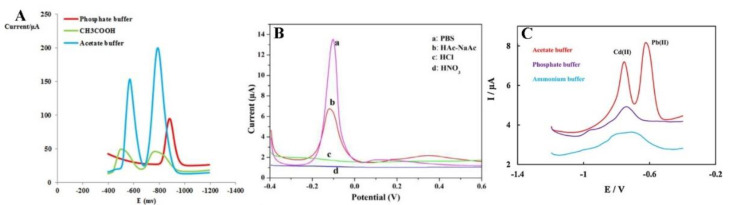
Electrolyte selection in determination of Pb^2+^ and Cu^2+^, by Dahaghin et al. [66] (**A**), Pb^2+^ by Wei et al. [55] (**B**), and Cd^2+^ and Pb^2+^ detection by Bagahayeri et al. [69] (**C**). All Figures are adapted from references [55,66,69] with permission from Elsevier.

Another extremely important parameter optimised during the analysis of metal ions is the selection of an appropriate electrolyte. The selection of a suitable supporting electrolyte and its pH guarantees the achievement of excellent electrochemical responses as well-formed, high-intensity current peaks. The electrolyte type affects the formation of various metals’ peaks (Figure 6). In heavy metal ions analysis, 0.1 M NaNO_3_ [56], 1 M HCl [60], and PBS [55] were selected, but a 0.1 M acetate buffer solution NaAc/HAc was the most commonly used and delivered the best results. The highest and best-defined peaks of metal ions are observed in the acetate buffer (Figure 6). The explanation of this phenomenon is complex and affected by many factors. Firstly, different electrodes may exhibit different electrochemical properties in the same electrolyte because an electrical double-layer is formed on the electrode as a result of an interaction between the cations or anions present in the solution. The double-layer model is described by many papers, including the Helmholtz, Gouy–Chapman, and Stern–Grahame models [74,75,76]. The authors of this review suggest that the increase of electrochemical signals of measured ions observed in the acetate buffer solution (NaAc/HAc) is caused not only by the appropriate pH but also because the acetate buffer enables the binding reaction as a result of intermolecular ion binding on the surface of the Fe_3_O_4_-modified electrode. The acetate buffer enables the reduction of ions by the intermolecular ion binding both the positively [55] and negatively charged species [69] and due to their interaction with the organic ligand [66]. Additionally, the authors suggest that the hydroxyl groups in the carboxylic group of acetic acid serve as active sites to adsorb heavy metal ions on the modified surface. 

**Figure 7:**
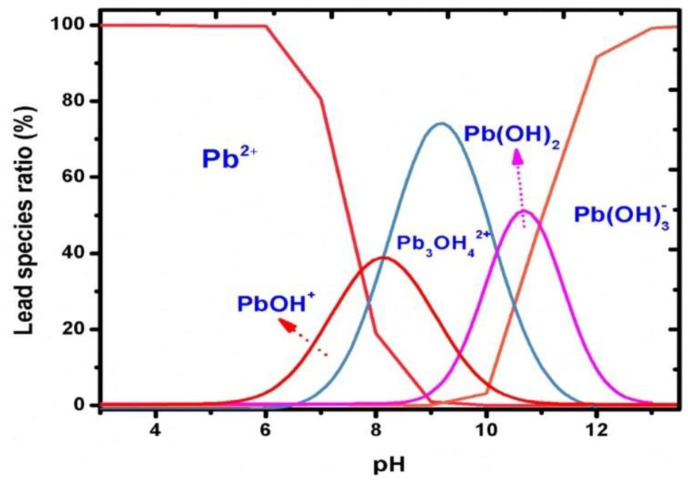
Distribution of the lead species as a function of pH presented by Qureashi et al. [56]. Figure is adapted from reference [56] with permission from Elsevier.

The pH value of the supporting electrolyte is the factor that inherently affects the intensity of the peaks of metal ions. The optimisation of the pH value is usually carried out in an acidic environment because above pH 7, the vast majority of metals form hydroxides, with the highest probability in the range of pH 4 and 6. However, the peaks with the highest intensity are obtained at pH 5 to 5.5 and less often within the range of pH 4 to 4.5. Qureashi et al. described the formation of Pb^2+^ ions occurring in a solution depending on the pH (Figure 7) [56]. 

Figure 7 shows that Pb^2+^ ions dominate in an acidic medium with a pH lower than 6. Subsequently, when the pH value increases to 8, the formation of a Pb(OH)^+^ complex occurs, and with a further increase to pH 9, the Pb_3_OH_4_^2+^ species is predominant. Pb(OH)_2_ shows maximum adsorption in a pH range from 9 to 11. The Pb(OH)_3_^−^ anionic complex exists in a pH range higher than 12 [56]. Similar relationships can be presented for other ions, for example Cd^2+^ [77] and Cu^2+^ [72]. 

Due to the ions species distribution, we can state that bi-positive ions adsorption experiments should be performed below pH 7. At the same time, an extremely low pH value may cause physiological changes in the Fe_3_O_4_ adsorbent. Based on these assumptions, the optimal pH for heavy metal ions analysis is in the range of pH 4 to 6.

The presence of magnetic nanoparticles on the GC electrode surface increases the sensor sensitivity even to the nano range. The electrode modification with bare Fe_3_O_4_ carried out by Fan et al. resulted in the development of a sensor with a limit of detection range of 119 nM, 154 nM, 83.9 nM, and 76.5 nM for Pb^2+^, Cd^2+^, Hg^2+^, and Cu^2+^, respectively [54]. The presence of functional groups on the nanomagnetite surface or additional modifiers increases the sensitivity even further. The highest sensitivity was reached by He et al., who created an electrochemical sensor by the immobilisation of Fe_3_O_4_/GN composite integrated with garlic extract (GE) onto the GC surface for the determination of Pb^2+^ in wastewater. The sensor exhibited two dynamic linear ranges including 0.001 to 0.5 nM and 0.5 to 1000 nM with an excellent low detection limit of 0.0123 pM (S/N = 3) and quantification limit (LOQ) of 0.41 pM (S/N = 10) [67]. A slightly lower sensitivity was obtained by Wu et al. [62], Kong, et al. [59], Baghayeri et al. [69], Dahaghin et al. [66], and Hu et al. [63] within tenths and hundredths of a nanomole for the variety of heavy metal ions. Among those mentioned in Table 1, the detection limit remained at the highest level for the sensors developed by Desmukh et al. They achieved LOD values in the range of 0.1, 0.05 μM, and 0.01 μM for individual analysis of Hg^2+^, Pb^2+^, and Cd^2+^ ions, respectively, whereas the LOD values for the simultaneous analysis of these ions were found to be 0.3 μM, 0.04 μM, and 0.2 μM, respectively, with the use of a sensor based on Fe_3_O_4_ nanoparticles capped with terephthalic acid [68].

## 4. Conclusions and Perspectives

This review covers a general discussion of trace metal electrochemical sensors based on the magnetic iron oxide, Fe_3_O_4_. Magnetite nanoparticles, which are a considerable part of the world of nanomaterials, have gained extensive significance in many fields of science. A multitude of applications has been progressed for the use of magnetite nanoparticles. Thanks to their properties, these nanoparticles are successfully used in many fields of chemistry, e.g., for remediation of contaminants using an external magnetic field, but are mainly used as electrode modifiers in electrochemistry. Modifiers based on Fe_3_O_4_ were used to create plenty of electrochemical sensors for various analytes detection, including heavy metal ions. This overview of recently published articles indicates that the GCE was the most commonly used conventional electrode surface for modification. However, SWASV and DPASW were the most commonly used measurement techniques for the detection of heavy metal ions.

In conclusion, the many possibilities and simplicity of the magnetite nanoparticles’ surface functionalisation provide the basis for the development of even more sensitive and selective sensors for heavy metal ions detection in the future. We can expect a possible development of commercial electrochemical sensors through the integration of standard electrodes with Fe_3_O_4_-based nanoparticles.

The Fe_3_O_4_ nanoparticles’ properties and the possibility of surface functionalisation are the foundation for obtaining new sensory platforms for a variety of analytes, not only for heavy metal ions detection. This phenomenon not only creates an opportunity to use nanomagnetite in many fields of chemistry but also in science in general.

## Figures and Tables

**Table 1:** Selected studies on Fe_3_O_4_ nanoparticles in electrochemical sensors for heavy metal ions detection.

Electrode	Method	Analyte	Detection limit		Ref
			Published	Converted	
Fe_3_O_4_@citrate/GCE	DPASV, CV	Pb^2+^	0.0061 μg·L^−1^	300 nM	[56]
Fe_3_O_4_/Bi_2_O_3_/C_3_N_4_/GCE	SWASV	Cd^2+^	3 × 10^−9^ mol·L^−1^	3 nM	[61]
		Pb^2+^	1 × 10^−9^ mol·L^−1^	1 nM	
Fe_3_O_4_@PDA@MnO_2_/mGCE	DPVSV	Pb^2+^	0.03 μg·L^−1^	0.14 nM	[60]
Fe_3_O_4_/F-MWCNTs/GCE	SWASV	Cd^2+^	0.05 nM	0.05 nM	[62]
		Pb^2+^	0.08 nM	0.08 nM	
		Cu^2+^	0.02 nM	0.02 nM	
		Hg^2+^	0.05 nM	0.05 nM	
Fe_3_O_4_/MWCNTs/LSG/CS/GCE	SWASV	Cd^2+^	0.1 μg·L^−1^	0.9 nM	[63]
		Pb^2+^	0.07 μg·L^−1^	0.3 nM	
Fe_3_O_4_/SiO_2_/CS/Nafion/GCE	DPASV	Cu^2+^	5 nmol·L^−1^	5 nM	[55]
GCE/GO/Fe_3_O_4_@PMDA/AuNPs	SWASV	As^3+^	0.15 ppb	2 nM	[64]
		Cu^2+^	0.11 ppb	2.4 nM	
Fe_3_O_4_@PANI/MGCE	DPASV	Cd^2+^	0.3 nmol·L^−1^	0.3 nM	[59]
		Pb^2+^	0.03 nmol·L^−1^	0.03 nM	
Fe_3_O_4_/F-MWCNTs/GCE	SWASV	Cd^2+^	0.014 μM	14 nM	[65]
		Pb^2+^	0.0084 μM	8.4 nM	
		Hg^2+^	0.0039 μM	3.9 nM	
		Zn^2+^	0.012 μM	12 nM	
		Cu^2+^	0.0053 μM	5.3 nM	
GO@Fe_3_O_4_@2-CBT/GCE	SWASV	Cd^2+^	0.03 ng·mL^−1^	0.27 nM	[66]
		Pb^2+^	0.02 ng·mL^−1^	0.1 nM	
Fe_3_O_4_/GCE	SWASV	Pb^2+^	0.119 μM	119 nM	[54]
		Cd^2+^	0.154 μM	154 nM	
		Hg^2+^	0.0839 μM	83.9 nM	
		Cu^2+^	0.0765 μM	76.5 nM	
DNA/Fe_3_O_4_@Au/MGCE	SWV	Ag^+^	3.4 nM	3.4 nM	[44]
		Hg^2+^	1.7 nM	1.7 nM	
Fe_3_O_4_@C/GCE	SWASV	Cd^2+^	40.9 nM	40.9 nM	[57]
		Pb^2+^	20.7 nM	20.7 nM	
		Cu^2+^	79.3 nM	79.3 nM	
NH_2_-Fe_3_O_4_@C/GCE	SWASV	Cd^2+^	23.1 nM	23.1 nM	
		Pb^2+^	28.5 nM	28.5 nM	
		Cu^2+^	38.4 nM	38.4 nM	
Fe_3_O_4_/GN/GE/GCE	SWASV	Pb^2+^	0.0123 pM	0.0123 pM	[67]
TA/Fe_3_O_4_/GCE	SWASV	Pb^2+^	0.04 μM	40 nM	[68]
		Hg^2+^	0.3 μM	300 nM	
		Cd^2+^	0.2 μM	200 nM	
GSH@Fe_3_O_4_/MGCE	SWASV	Pb^2+^	0.182 μg·L^−1^	0.9 nM	[69]
		Cd^2+^	0.172 μg·L^−1^	1.5 nM	

## Data Availability

No new data were created or analysed in this study. Data sharing is not applicable to this article.

## Abbreviations

2-CBT	benzothiazole-2-carboxaldehyde
ASV	anodic stripping voltammetry
CS	chitosan
CSV	cathodic stripping voltammetry
CV	cyclic voltammetry
DMF	*N*,*N*-Dimethylformamide
DPASV	differential pulse anodic stripping voltammetry
DPV	differential pulse voltammetry
F-MWCNTs	fluorinated multi-walled carbon nanotubes
GCE	glassy carbon electrode
GE	garlic extract
GN	graphene
GO	graphene oxide
GSH	glutathione
HDME	hanging drop mercury electrode
IPA	isopropyl alcohol
LOD	limit of detection
LOQ	limit of quantification
LSG	laser scribed graphene
mGCE, MGCE	magnetic glassy carbon electrode
MWCNTs	multi-walled carbon nanotubes
NaAc/HAc	acetate buffer solution
NPs	nanoparticles
PANI	polyaniline
PDA	polydopamine
PMDA	poly methyldopa
S/N	signal to noise ratio
SWAdCSV	square wave adsorptive cathodic stripping voltammetry
SWASV	square wave anodic stripping voltammetry
SWV	square wave voltammetry
TA	terephthalic acid
PDA	polydopamine
PMDA	poly methyldopa
SWAdCSV	square wave adsorptive cathodic stripping voltammetry
SWASV	square wave anodic stripping voltammetry
TA	terephthalic acid
